# Self-sampling for HPV genotyping: a study of vaginal and urine collection in Brazilian women with high-grade lesions

**DOI:** 10.1016/j.clinsp.2025.100780

**Published:** 2025-09-17

**Authors:** Cristina Paula Castanheira, Noely Paula Cristina Lorenzi, Fernanda Dahrouge Chiarot, Alex Jones Flores Cassenote, Maricy Tacla, Adhemar Longatto-Filho, José Maria Soares-Junior, Edmund C. Baracat, Luisa Lina Villa, Gustavo A.R. Maciel, Lara Termini

**Affiliations:** a Department of Obstetrics and Gynecology, Hospital das Clínicas, Faculdade de Medicina, Universidade de São Paulo (HC-FMUSP), São Paulo, SP, Brazil; b Department of Obstetrics and Gynecology, Conjunto Hospitalar Mandaqui, São Paulo, SP, Brazil; c Department of Gynecology, Hospital Universitario da Universidade de São Paulo, São Paulo, SP, Brazil; d Gynecologic Oncology Research Institute IPOGLab, São Paulo, SP, Brazil; e Instituto do Cancer do Estado de São Paulo (ICESP), Hospital das Clínicas, Faculdade de Medicina, Universidade de São Paulo (HC-FMUSP), São Paulo, SP, Brazil; f Preventive Medicine Department, Universidade de São Paulo (USP), São Paulo, SP, Brazil; g Life and Health Science Research Institute (ICVS), School of Medicine, University of Minho, Braga, Portugal; h Laboratory of Medical Investigation (LIM14), Faculdade de Medicina, Universidade de São Paulo (FMUSP), São Paulo, SP, Brazil; i Structural and Molecular Gynecology Laboratory (LIM-58), Gynecology Discipline, Department of Obstetrics and Gynecology, Hospital das Clínicas, Faculdade de Medicina, Universidade de São Paulo (HC-FMUSP), São Paulo, SP, Brazil; j Comprehensive Center for Precision Oncology, Universidade de São Paulo (USP), São Paulo, SP, Brazil; k Grupo Fleury, São Paulo, SP, Brazil

**Keywords:** Primary HPV screening, Human papillomavirus, Self-sampling, First-void urine, Cervical cancer screening

## Abstract

**Objective:**

In Brazil — a country of continental dimensions with marked socioeconomic disparities — the use of self-collected samples and first-void urine for cervical cancer screening may be particularly valuable. This study aimed to assess the acceptability of two self-sampling approaches - first-void urine collection and vaginal self-sampling - among women diagnosed with high-grade cervical lesions (CIN2+) referred to a tertiary care center. Additionally, the study evaluated the concordance of high-risk HPV (hrHPV) test results obtained from self-collected samples compared to those collected by a healthcare professional.

**Methods:**

This cross-sectional study included 100 women. Participants completed a structured questionnaire on clinical history, demographics, gynecological and obstetric background. Following an instructional video, they performed self-collection of urine and vaginal samples. All participants then underwent colposcopic examination for lesion assessment and therapeutic planning. HPV DNA testing was conducted, and agreement analysis was performed between sample types.

**Results:**

Both urine and vaginal self-collection methods were reported as easy and comfortable. Instructions were considered easy or very easy by nearly all participants for all collection methods. Clinician-collected sampling was associated with higher embarrassment and discomfort. Agreement analysis showed excellent concordance for HPV 16 and other high-risk HPV types between self-collected, urine, and clinician-collected samples, with all comparisons reaching statistical significance.

**Conclusions:**

Urine and vaginal self-collection are feasible, acceptable, and reliable methods. Urine sampling was the preferred method in the present study. High concordance with clinician-collected samples confirms their clinical utility, and the positive response to instructional videos highlights the importance of educational support.

## Highlights

• Urine and vaginal self-sampling were highly acceptable among women with CIN2+.
• Instructional videos were effective and well-received for self-collection guidance.
• HPV testing showed excellent agreement, especially for HPV16 detection.
• Findings support self-collection in national programs to expand screening coverage.

## Introduction

Cervical Cancer (CC) remains a significant public health challenge, particularly in developing countries like Brazil. The Brazilian National Cancer Institute (INCA) estimates approximately 17,010 new cases for the 2023–2025 triennium, representing the third most common cancer among women in this country.^1^ In Brazil, cervical cancer screening is conducted opportunistically, using Pap cytology in women aged 25 to 64 years.^2^ Organized cytology-based screening programs have been effective in reducing cervical cancer incidence and mortality rates. However, the literature highlights important limitations of this method, including its low sensitivity and challenges related to the standardization and quality control of cervical cytology.^3^^,^^4^

Brazil is a country of continental dimensions with significant socioeconomic disparities and cultural and structural barriers to accessing healthcare services. Combined with cervical cancer incidence and mortality rates, this scenario favors the adoption of a more sensitive screening method to control cervical cancer.

In March 2024, the Brazilian Ministry of Health published guidelines authorizing the use of molecular tests for high-risk oncogenic human Papillomavirus (hrHPV) as primary screening for cervical cancer. This guideline, to be implemented by the end of 2025, establishes that cervical cancer screening in Brazil must be organized, using molecular hrHPV testing followed by cytology when necessary.^5^, ^6^, ^7^ Molecular tests for the detection of hrHPV have proven superior to conventional cytology, offering greater sensitivity for identifying invasive cancers, precursor lesions, and adenocarcinomas, with a 60 %‒70 % increase in abnormality detection.^8^, ^9^, ^10^, ^11^, ^12^ In addition, these tests offer greater cost-effectiveness, mainly due to their high negative predictive value, which enables the extension of intervals between exams in case of negative results.^9^^,^^13^

Limited access to healthcare services, the need for well-trained healthcare teams to assist women, and cultural factors such as fear or shame, pain, and physical limitations also hinder the effectiveness of the Brazilian cervical cancer screening program. Due to their less invasive nature and the possibility of being performed outside clinical settings, vaginal self-sampling devices have significant potential to improve screening effectiveness.^14^^,^^15^ Different studies demonstrate the good acceptability of vaginal and urine self-sampling devices, which can increase women's participation in cervical cancer prevention programs.^16^^,^^17^ hrHPV detection tests have shown similar performance in self-collected vaginal samples compared to samples collected by healthcare professionals for the detection of cervical cancer and its precursor lesions. Several countries, such as the Netherlands and Australia, have already incorporated this collection method into their screening programs.^18^^,^^19^

The use of First-Void Urine (FVU) samples for molecular hrHPV testing has also proven to be an effective and well-accepted alternative among women.^20^, ^21^, ^22^, ^23^ Besides, urine collection is justified by its ease of use and high acceptability, especially among women who report discomfort or fear regarding vaginal self-sampling. This is extremely important as it may increase screening adherence by providing a non-invasive, easily applicable method with the potential to expand access among populations with low adherence to traditional screening methods.^24^^,^^25^

In this context, the use of educational videos by healthcare services emerges as an important tool to support the understanding of health-related information.^26^^,^^27^ In the present study, an instructional video was presented to enhance participants' understanding of the self-collection process, explaining the different sampling methods.

Therefore, the objective of this study was to evaluate the acceptability of urine collection using the Colli-Pee® device and vaginal self-sampling with the Coari® brush, in comparison to the collection performed by a healthcare professional. Moreover, the authors aimed to assess the acceptability of urine sampling as an alternative method for cervical cancer screening among women with high-grade cervical lesions (CIN2+) referred for treatment at a tertiary care center. Finally, the study also evaluated the agreement of hrHPV test results between the different sample collection methods.

## Methods and methods

### Study population

This was a cross-sectional study involving 100 women aged 21-years and older referred from Basic Health Units to a tertiary healthcare facility, the Clinics Hospital of Faculdade de Medicina, Universidade de São Paulo, in São Paulo, Brazil, for colposcopy due to abnormalities detected in cervical cytology or histopathological biopsy findings. Samples were collected between January and September 2024. The exclusion criteria were pregnancy, prior total hysterectomy, immunosuppression, or a history of previous treatment for HPV-induced cervical lesions. All participants signed an informed consent form (Supp. Data 1). The volunteers completed a questionnaire covering clinical information, demographic data, gynecological and obstetric history, sexual practices, and comorbidities. Subsequently, the participants watched an instructional video explaining the devices used for urine collection (Colli-Pee®) and vaginal self-sampling (Coari® brush).

### Sample collection procedures

After watching the instructional video, the participants received verbal instructions on the use of the Colli-Pee® FV-500 device (Novosanis®, Wijnegem, Belgium) as well as the Coari® vaginal self-sampling brush (Kolplast®, São Paulo, Brazil). Sequential collection of urine, vaginal self-sample, and clinician-collected cervical samples was then performed. The Colli-Pee® FV-500 device standardizes the collection of first void urine (13–20 mL), which is rich in cells from the lower genital tract, improving the sensitivity and reliability of molecular tests. Participants collected the urine sample in the restroom and returned the device to the healthcare staff. Vaginal self-sampling with the Coari® brush was then performed either in the examination room or in the restroom, based on participant preference. The procedure involved inserting the Coari® brush into the vagina, rotating it five times, and then removing it. The used brush was handed to the healthcare professional, who immediately added 19 mL of cell preservation solution (CellPreserv®, Kolplast®). Subsequently, all participants underwent colposcopic examination to evaluate cervical lesions and determine therapeutic management. Prior to colposcopy, cervical samples were collected using a sterile cervical brush (Kolplast®), which was inserted into the external cervical orifice and rotated five times. The material was immediately placed in cell preservation solution (CellPreserv®, Kolplast®). After the sequential collection of all three samples, participants completed a questionnaire assessing the acceptability of each sampling method as well as their satisfaction with the instructional video (Supp. Data 2). Immediately after collection, samples were transported to the laboratory, aliquoted, and stored at −20 °C for subsequent analysis.

### HPV DNA testing

HPV DNA testing was performed using the Cobas® 4800 HPV Test at the Laboratory of the Gynecologic Oncology Research Institute (IPOG), according to the manufacturer’s instructions. The Cobas HPV test provides specific genotyping for HPV16 and HPV18, as well as pooled results for 12 other high-risk types: HPV31, HPV33, HPV35, HPV39, HPV45, HPV51, HPV52, HPV56, HPV58, HPV59, HPV66, and HPV68. Sample preparation was conducted using the Cobas X480 platform, which simultaneously extracts, purifies, and prepares DNA for HPV and β-globin amplification.

### Instructional video

An instructional video approximately four minutes in length was shown to participants, providing instructions on how to handle and use the vaginal and urine self-sampling devices (https://www.youtube.com/watch?v=387uySsByHI).

### Sample size calculation and statistical methods

Sample size was calculated assuming an estimated HPV prevalence of 50 %, which maximizes sample size under a binomial distribution, with a 10 % absolute margin of error and a 95 % confidence level (α = 0.05). Based on this assumption, a minimum of 97 participants was required. Descriptive statistics were used to summarize sociodemographic, clinical, and reproductive characteristics of the participants. Categorical variables were expressed as absolute and relative frequencies with 95 % Confidence Intervals (95 % CI). Continuous variables were summarized using means and Standard Deviations (SD). Participants’ responses regarding acceptability and preferences for each sampling method were also presented as proportions with 95 % CIs. HPV DNA positivity rates by type (any HPV, HPV16, HPV18, and Other High-Risk types – OHR) were calculated for each sample collection method (urine, vaginal self-sampling, and clinician-collected cervical samples). Comparisons between methods were expressed as proportions with 95 % CIs and illustrated graphically. To assess the concordance of HPV detection across collection methods, Cohen’s Kappa coefficient (κ) was calculated for each HPV type. Agreement was interpreted according to Landis and Koch’s criteria.^28^ slight (≤ 0.20), fair (0.21–0.40), moderate (0.41–0.60), substantial (0.61–0.80), and almost perfect (≥ 0.81). All statistical tests were two-tailed and statistical significance was set at *p* < 0.05. All analyses were performed using IBM® SPSS® Statistics, version 27 (IBM Corp., Armonk, NY, USA) and *R* software (*R* Foundation for Statistical Computing, Vienna, Austria), version 4.4.0.

### Ethical considerations

All study procedures were conducted in accordance with the standards of the Institutional Ethics Committee under number CAAE: 55,640,222.7.0000.0068. All participants received a detailed explanation of the study objectives and procedures, and signed an informed consent form.

## Results

The study population comprised 100 women diagnosed with high-grade cervical lesions. The majority of participants were between 30- and 39-years of age (39.4 %), followed by those aged 40–49 years (25.3 %) and 20–29 years (19.2 %). With regard to self-reported race/ethnicity, 56.0 % identified as Brown or mixed race, 26.0 % as White, and 17.0 % as Black. In terms of educational level, 62.0 % had completed secondary education, and 13.0 % reported having higher education. Most participants were either married or in a stable union (53.0 %). Regarding religious affiliation, 45.0 % identified as Evangelical or Protestant, while 28.0 % reported being Catholic. The majority of participants were non-smokers (83.0 %) (Table 1).

**Table 1:** Sociodemographic and clinicopathological characteristics of the study participants.

	N	% (95 % CI)
**Age**		
20–29 years	19	19.2 (12.4‒27.8)
30–39 years	39	39.4 (30.2‒49.2)
40–49 years	25	25.3 (17.5‒34.4)
50–59 years	11	11.1 (6.0‒18.4)
60 years and over	5	5.1 (2.0‒10.7)
**Self-reported race/ethnicity**		
White	26	26.0 (18.2‒35.2)
Black	17	17.0 (10.6‒25.2)
Brown (Mixed)	56	56.0 (46.2‒65.4)
Indigenous	1	1.0 (0.1‒4.6)
**Educational level**		
Primary education	25	25.0 (17.3‒34.1)
Secondary education	62	62.0 (52.3‒71.1)
Higher education	13	13.0 (7.5‒20.6)
**Marital status**		
Married / Stable Union	53	53.0 (43.2‒62.6)
Divorced	10	10.0 (5.3‒17.0)
Single	34	34.0 (25.3‒43.6)
Widowed	3	3.0 (0.9‒7.8)
**Religion affiliation**		
Catholic	28	28.0 (19.9‒37.3)
Evangelical / Protestant	45	45.0 (35.5‒54.8)
Other Religions	4	4.0 (1.4‒9.2)
No Religion	23	23.0 (15.6‒31.9)
**Smoking habit**		
No	83	83.0 (74.8‒89.4)
Yes	17	17.0 (10.6‒25.2)
**Contraceptive method**		
Hormonal methods	37	37.0 (28.0‒46.7)
Intrauterine device (IUD)	4	4.0 (1.4‒9.2)
Definitive Contraceptions Methods^c^	14	14.0 (8.3‒21.8)
Barrier methods (condoms)	14	14.0 (8.3‒21.8)
No contraceptive method	31	31.0 (22.6‒40.5)
**Number of pregnancies**		
No pregnancies	9	9.0 (4.5‒15.8)
1 pregnancy	20	20.0 (13.1‒28.6)
2 to 4 pregnancies	53	53.0 (43.2‒62.6)
≥ 5 pregnancies	18	18.0 (11.4‒26.4)
**Number of births**		
No births	12	12.0 (6.7‒19.4)
1 birth	20	20.0 (13.1‒28.6)
2 to 4 births	58	58.0 (48.2‒67.3)
≥ 5 births	10	10.0 (5.3‒17.0)
**Number of abortions**		
No abortions	67	67.0 (57.4‒75.6)
1 abortion	22	22.0 (14.8‒30.8)
2 to 4 abortions	11	11.0 (6.0‒18.2)
**Initial cervical cytology result**		
Negative for Intraepithelial Lesion or Malignancy (NILM)	3	3.0 (0.9‒7.8)
Low-grade Squamous Intraepithelial Lesion (LSIL)	3	3.0 (0.9‒7.8)
Atypical Squamous Cells (ASC) ‒ US	2	2.0 (0.4‒6.3)
Atypical Squamous Cells (ASC) ‒ H	17	17.0 (10.6‒25.2)
High-grade Squamous Intraepithelial Lesion (HSIL)^a^	3	3.0 (0.9‒7.8)
High-grade Squamous Intraepithelial Lesion (HSIL)	71	71.0 (61.6‒79.2)
Adenocarcinoma in situ	1	1.0 (0.1‒4.6)
**Cervical biopsy result**		
Low-grade lesion (CIN1)^b^	1	1.0 (0.1‒4.6)
High-grade lesion (CIN2)	51	51.0 (41.3‒60.7)
High-grade lesion (CIN3)	47	47.0 (37.4‒56.8)
Adenocarcinoma in situ	1	1.0 (0.1‒4.6)
**Histological results of the excised transformation zone specimen**		
NILM (Chronic cervicitis)	13	13.0 (7.5‒20.6)
Low-grade lesion (CIN1)	8	8.0 (3.9‒14.5)
High-grade lesion (CIN2)	15	15.0 (9.0‒23.0)
High-grade lesion (CIN3)	57	57.0 (47.2‒66.4)
Invasive squamous cell carcinoma	1	1.0 (0.1‒4.6)
Invasive adenocarcinoma	1	1.0 (0.1‒4.6)
Squamous cell carcinoma with superficial stromal invasion	2	2.0 (0.4‒6.3)
Awaiting surgical excision of the cervical transformation zone	3	3.0 (0.9‒7.8)

a Microinvasion cannot be ruled out.

b The participant underwent an excisional procedure due to HSIL detected on cytology.

c Tubal ligation/vasectomy.

Reproductive history showed that 53.0 % of participants had experienced two to four pregnancies, and 58.0 % had given birth two to four times. A total of 33.0 % reported at least one abortion, with no participant reporting more than two. Hormonal contraceptives were the most frequently used method (37.0 %), while 31.0 % reported using no contraceptive method. As expected, due to the study’s inclusion criteria, the majority of participants presented high-grade abnormalities on initial cytology, including 71.0 % HSIL and 17.0 % ASC—H. Cervical biopsy results confirmed the presence of high-grade lesions in the majority of participants, with 51.0 % diagnosed with Cervical Intraepithelial Neoplasia grade 2 (CIN2) and 47.0 % with grade 3 (CIN3). Histopathological analysis of the surgical specimens revealed CIN2 or CIN3 in 72.0 % of the cases. Additionally, two participants were diagnosed with squamous cell carcinoma exhibiting superficial stromal invasion, while two others presented with invasive carcinoma ‒ one squamous and the other adenocarcinoma. Conversely, chronic cervicitis was identified in 13.0 % of the cases.

As shown in Table 2, participants reported high levels of ease and comfort with both self-collection devices. Regarding the urine collection device, all participants considered the instructions to be either very easy (51.0 %) or easy (49.0 %), and its use was similarly rated as very easy (48.0 %) or easy (52.0 %). Reports of embarrassment and discomfort during urine collection were minimal, with 97.0 % of participants indicating no embarrassment and 97.0 % reporting no discomfort.

**Table 2:** Participants’ experience, preferences, and perceptions regarding sample collection methods and the educational video in the HPV self-collection acceptability study.

	n	% (95 % CI)
**Instruction on the use of the urine collector**		
Very easy	51	51.0 (41.3‒60.7)
Easy	49	49.0 (39.3‒58.7)
**Use of the urine collector**		
Very easy	48	48.0 (38.4‒57.7)
Easy	52	52.0 (42.3‒61.6)
**Embarrassment or discomfort when using the urine collector**		
Not at all embarrassed	97	97.0 (92.2‒99.1)
Slightly embarrassed	3	3.0 (0.9‒7.8)
**Discomfort or pain when using the urine collector**		
No discomfort at all	97	97.0 (92.2‒99.1)
Slight discomfort	3	3.0 (0.9‒7.8)
**Instruction for using the self-collection device**		
Very easy	41	41.0 (31.7‒50.8)
Easy	56	56.0 (46.2‒65.4)
Difficult	3	3.0 (0.9‒7.8)
**Use of the self-collection device**		
Very easy	37	37.0 (28.0‒46.7)
Easy	59	59.0 (49.2‒68.3)
Difficult	4	4.0 (1.4‒9.2)
**Embarrassment or discomfort when using the self-collection device**		
Not at all embarrassed	88	88.0 (80.6‒93.3)
Slightly embarrassed	10	10.0 (5.3‒17.0)
Moderately embarrassed	2	2.0 (0.4‒6.3)
**Instruction regarding sample collection by the healthcare professional**		
Very easy	38	38.0 (28.9‒47.7)
Easy	61	61.0 (51.2‒70.1)
Difficult	1	1.0 (0.1‒4.6)
**Embarrassment during sample collection by the healthcare professional**		
Not at all embarrassed	72	72.0 (62.7‒80.1)
Slightly embarrassed	24	24.0 (16.4‒33.0)
Moderately embarrassed	2	2.0 (0.4‒6.3)
Very embarrassed	2	2.0 (0.4‒6.3)
**Discomfort during sample collection by the healthcare professional**		
No discomfort at all	63	63.0 (53.3‒72.0)
Slight discomfort	28	28.0 (19.9‒37.3)
Moderate discomfort	6	6.0 (2.5‒11.9)
Severe discomfort	3	3.0 (0.9‒7.8)
**Choice of collection method**		
Urine collection device	53	53.0 (43.2‒62.6)
Self-collected device (vaginal brush)	17	17.0 (10.6‒25.2)
Professional collection	14	14.0 (8.3‒21.8)
No preference / Indifferent	16	16.0 (9.8‒24.1)
**Reasons for choosing the collection method**		
Greater convenience	54	54.0 (44.2‒63.5)
Possibility of collecting the sample alone	55	55.0 (45.2‒64.5)
Ease of collecting the sample at home	51	51.0 (41.3‒60.7)
Less embarrassment or shame	20	20.0 (13.1‒28.6)
Less discomfort or pain	26	26.0 (18.2‒35.2)
Fear of incorrect self-collection	14	14.0 (8.3‒21.8)
Greater trust in collection by health professional	15	15.0 (9.0‒23.0)
Indifferent – accepts all three methods	15	15.0 (9.0‒23.0)
**Opinion about the educational video**		
Helped very little	1	1.0 (0.1‒4.6)
Helped very much	99	99.0 (95.4‒99.9)
**Healthcare services should offer more educational videos**	100	100.0
**Reasons for approving or disapproving the educational video**		
I would like to watch the video and clarify my doubts with a health professional	38	38.0 (28.9‒47.7)
The video made me feel calmer and safer to use the urine collector and the brush	62	62.0 (52.3‒71.1)

For the vaginal self-collection device, the instructions were rated as very easy by 41.0 % of participants and as easy by 56.0 %, with only 3.0 % indicating any difficulty. The use of the device was described as very easy or easy by 96.0 % of respondents. Most participants reported no embarrassment (88.0 %) and only slight discomfort, while moderate embarrassment was reported by a small minority (2.0 %).

When evaluating clinician-collected sampling, although the instructions were perceived as “very easy” by 38.0 % and “easy” by 61.0 % of participants, higher levels of embarrassment and discomfort were reported compared to self-collection methods. While 63.0 % of participants reported no discomfort during clinician collection, 28.0 % experienced mild discomfort, and 10.0 % reported moderate to severe embarrassment. Overall, urine collection was the most preferred method (53.0 %), followed by self-collection (17.0 %) and clinician collection (14.0 %). The most frequently cited reasons for these preferences included convenience (54.0 %), the ability to collect the sample independently (55.0 %), and the possibility of performing the procedure at home (51.0 %). The instructional video was overwhelmingly well evaluated: 99.0 % of participants reported that it facilitated understanding of the procedures, and all agreed that healthcare services should offer instructional videos. Furthermore, 62.0 % indicated that watching the video made them feel more confident and secure when using the devices. Conversely, 38.0 % stated that, although they found the video helpful, they would prefer to have a healthcare professional available to clarify any doubts after watching the video. tFig. 1 illustrates the HPV positivity rates by type and collection method. Detection of any HPV was highest in samples collected by clinician 86.0 % (95 % CI: 78.2 %–91.7 %), followed by self-collection 82.0 % (95 % CI: 73.6 %–88.6 %) and urine collection 73.0 % (95 % CI: 63.7 %–81.0 %). HPV16, positivity was similar in the three methods: 39.0 % (95 % CI: 29.9 %–48.8 %) for clinician, 40.0 % (95 % CI: 30.8 %–49.8 %) for self-collection, and 37.0 % (95 % CI: 28.0 %–46.7 %) for urine samples. HPV18 was detected in only a small fraction of participants, with 3.0 % positivity in both urine (95 % CI: 0.9 %–7.8 %) and clinician-collected sampling (95 % CI: 0.9 %–7.8 %), and 4.0 % in self-collected samples (95 % CI: 1.4 %–9.2 %). Other high-risk HPV (OHR), detection was more frequent in health professional collection 61.0 % (95 % CI: 51.2 %–70.1 %), followed by self-collection 57.0 % (95 % CI: 47.2 %–66.4 %) and urine 49.0 % (95 % CI: 39.3 %–58.7 %).

**Fig. 1:**
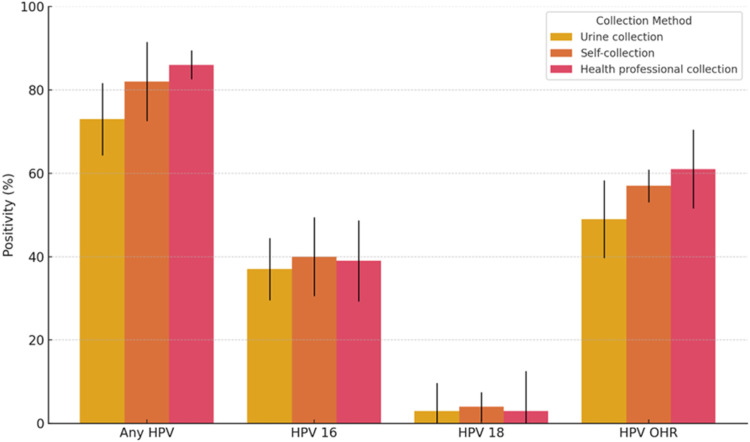
HPV positivity by type and sample collection method among participants with high-grade cervical lesions in the HPV self-collection acceptability study.

In Table 3, it is possible to observe the kappa values indicating the level of agreement between the different sample collection methods for each HPV type. For HPV16, kappa values ranged from 0.937 to 0.979, indicating excellent agreement between urine, self-collection, and health professional collection methods. Agreement was also high for any HPV (κ = 0.611–0.780) and HPV OHR (κ = 0.761–0.876), with all comparisons showing statistically significant results (*p* < 0.001). For HPV18, although less frequent, moderate to strong agreement was observed (κ = 0.656–0.852), with slightly lower performance involving professional collection. These findings confirm the consistency of alternative collection methods for HPV detection in women with high-grade cervical lesions.

**Table 3:** Agreement between sample collection methods for HPV detection by type among participants with high-grade cervical lesions in the HPV self-collection acceptability study.

Method 1	Method 2	Concordant		Discordant		Kappa	p-value
		+	–	+ → -	- → +		
**Any HPV**							
Urine	Self-collection	73	18	0	9	0.745	<0.001
Urine	Health professional collection	73	14	0	13	0.611	<0.001
Self-collection	Health professional collection	81	13	1	5	0.780	<0.001
**HPV16**							
Urine	Self-collection	37	60	0	3	0.937	<0.001
Urine	Health professional collection	37	61	0	2	0.958	<0.001
Self-collection	Health professional collection	39	60	1	0	0.979	<0.001
**HPV18**							
Urine	Self-collection	3	96	0	1	0.852	<0.001
Urine	Health professional collection	2	96	1	1	0.656	0.006
Self-collection	Health professional collection	3	96	1	0	0.852	<0.001
**HPV OHR**							
Urine	Self-collection	49	43	0	8	0.840	<0.001
Urine	Health professional collection	49	39	0	12	0.761	<0.001
Self-collection	Health professional collection	56	38	1	5	0.876	<0.001

## Discussion

This study evaluated the acceptability, feasibility, and diagnostic agreement of urine and vaginal self-collection devices for HPV genotyping among women diagnosed with high-grade cervical lesions. The findings support the use of self-collection as a reliable and well-accepted alternative to clinician-based sampling and offer relevant insights for optimizing cervical cancer screening strategies, especially in underserved populations.

One of the main findings of this study was the high feasibility and acceptability of self-collection methods among women with high-grade cervical lesions. Notably, 70 % of participants chose self-collection over clinician collection as their preferred method, particularly with urine self-collection, where 97 % of participants reported no discomfort. Furthermore, 100 % and 97 % of participants found urine and vaginal self-collection devices easy to use, respectively. These findings align with earlier research and reinforce the potential of self-collection for use in high-risk populations where structural and psychosocial barriers persist.^29^^,^^30^

Urine collection, in particular, emerged as the most preferred method (57 %), with participants citing convenience, autonomy, and the possibility of home-based use as important advantages. These findings are consistent with other reports.^31^^,^^32^ and have significant implications for public health strategies aiming to expand HPV screening coverage through non-invasive and user-friendly approaches. In resource-limited or culturally restrictive settings, urine sampling with devices like Colli-Pee® may represent an ideal solution to increase participation, particularly in hard-to-reach or vulnerable populations.

The diagnostic performance of self-collection was also robust. High-risk HPV was detected in 86 % of clinician-collected samples, 82 % of vaginal self-collected samples, and 73 % of urine samples. Genotype-specific analysis revealed similar detection rates of HPV16 across all methods: 39 % (clinician), 40 % (vaginal self-collection), and 37 % (urine), highlighting the reliability of both self-collection approaches for detecting the most oncogenic HPV type.

Cohen’s kappa analysis further supported these findings, showing almost perfect agreement for HPV16 detection and substantial agreement for overall hrHPV detection. These results mirror systematic reviews reporting high concordance between self- and clinician-collected samples in high-risk populations and validate the use of self-collection in diagnostic workflows.^33^ Future studies involving larger cohorts are needed to further investigate genotype-specific agreement and evaluate potential variability across different population subgroups.

Another strength of this study was the use of an instructional video to instruct participants on the correct use of self-collection devices. Nearly all participants (99 %) reported that the video was helpful, and 100 % believed such resources should be routinely available in healthcare services. These findings emphasize the role of digital educational tools in enhancing comprehension, reducing anxiety, and improving adherence to self-collection protocols. Previous studies have shown that audiovisual materials can significantly boost health literacy and screening participation.^26^ The incorporation of such tools into national screening programs could greatly facilitate the scale-up of self-collection-based interventions, particularly in low-literacy or remote settings.

These results are especially relevant in the context of Brazil’s transition to HPV DNA testing as the primary cervical cancer screening strategy. Given the country’s vast geography and socioeconomic inequalities, integrating self-collection ‒ especially urine collection ‒ into screening protocols may help overcome logistical and cultural barriers that limit participation. Previous modeling studies have shown that a single HPV DNA test at age 35 can reduce a woman’s lifetime risk of cervical cancer by approximately 25 % to 36 %.^34^ When paired with accessible self-collection technologies and educational interventions, this approach could substantially improve coverage, especially among underserved populations such as Indigenous communities, incarcerated individuals, people with disabilities, and LGBTQIA+ groups.^35^

Despite its strengths, this study has limitations. The sample included only women with histologically confirmed high-grade lesions, which limits the generalizability of the findings to the general screening population. Additionally, because participants were not instructed to avoid genital hygiene before collection, HPV detection rates may have been affected, particularly in urine samples. Finally, collections were conducted in a hospital setting with a professional available to clarify doubts ‒ this may have positively influenced both the acceptability and correct use of the devices. Future community-based studies should assess the performance of self-collection methods as used routinely and without supervision.

## Conclusion

This study demonstrates that both urine and vaginal self-collection are feasible, acceptable, and diagnostically reliable methods for HPV detection in women with high-grade cervical lesions. Urine self-collection was the preferred method, highlighting its potential as a non-invasive and user-friendly option for expanding screening access. High concordance with clinician-collected samples confirms the clinical utility of these approaches. Furthermore, the strong positive response to instructional videos underscores the value of integrating educational tools to support self-collection and increase participation. These findings emphasize the value of adopting self-collection approaches within Brazil’s national screening framework.

## Ethical considerations

All study procedures were conducted in accordance with the standards of the Institutional Ethics committee under number CAAE: 55640222.7.0000.0068). All participants received a detailed explanation of the study objectives and procedures, and signed an informed consent form.

## Declaration of generative AI and AI-assisted technologies in the writing process

During the development of this manuscript, the authors used ChatGPT (OpenAI) to enhance the clarity, readability, and linguistic quality of the text. All content generated with the assistance of this tool was thoroughly reviewed, edited, and approved by the authors, who assume full responsibility for the final version of the manuscript.

## Submission declaration

This work has not been previously published. It is not under consideration for publication elsewhere. All authors approve of the article.

## Funding

This work was supported by Fundação de Amparo à Pesquisa do Estado de São Paulo/FAPESP (grant number 2022/16783–3) and Grupo Fleury (grant number NP-679).

## Conflicts of interest

The authors declare that they have no known competing financial interests or personal relationships that could have appeared to influence the work reported in this paper.

## Appendix Supplementary materials

Image, application 1
