# Supplementary material for: Self-sampling for HPV genotyping: a study of vaginal and urine collection in Brazilian women with high-grade lesions
Source: Clinics (Sao Paulo). 2025 Sep 17;80:100780. doi: 10.1016/j.clinsp.2025.100780 (PMC12478266; doi:10.1016/j.clinsp.2025.100780)
Supplement: Supplementary file 1 [file mmc1.docx]

**CLINICS-D-25-01348_ Supplementary material**

**Supplementary Data 1 ICF**

**HOSPITAL DAS CLÍNICAS, SCHOOL OF MEDICINE, UNIVERSITY OF SÃO PAULO – HCFMUSP**
**INFORMED CONSENT FORM**

**Study Title – COMPARATIVE ANALYSIS OF THE PRESENCE OF HPV, MICROBIOTA COMPONENTS, AND GENE METHYLATION PROFILE IN CERVICAL CELLS OBTAINED BY URINE, SELF-COLLECTION, AND HEALTHCARE PROFESSIONALS**

**Principal Investigator – Gustavo Arantes Rosa Maciel**

**Department/Institute – Division of Gynecology, Department of Obstetrics and Gynecology, School of Medicine, University of São Paulo / Central Institute of the Hospital das Clínicas, FMUSP (ICHC-FMUSP)**

You are being invited to voluntarily participate in a study that will be conducted at the Gynecology Outpatient Clinic of the Hospital das Clínicas of the University of São Paulo School of Medicine. The study will include women referred for treatment due to cervical biopsy abnormalities. It is important that you read and understand all procedures and ensure that all your questions have been answered before signing this document. This study is important in our effort to identify simpler methods for performing routine cervical cancer screening. Your treatment does not depend on your participation in this study, although your participation would be appreciated.

If you agree to participate, you will be one of 100 participants, along with 99 other women. During your avaliation, you should inform the doctor about any current diseases and treatments you have had or are currently undergoing.

Detailed data will be collected regarding your avaliation, medications used, and gynecological and obstetric history. All participants will undergo a physical and gynecological examination, and it may be necessary for you to undergo a colposcopy (a more detailed Pap smear examination using a microscope). Before the gynecological and colposcopy exams, you will collect your urine in a container and perform a self-sampling Pap smear using a small brush (self-collection). This self-collection of vaginal samples will be done using a soft brush developed for self-use. This procedure is generally painless, though you may feel slight discomfort. After that, a healthcare professional will collect a cervical sample; this collection may cause mild discomfort but does not pose any risk to participants.

After this sample collection, participants may need to undergo a colposcopy exam. If necessary, during this procedure, a small tissue sample (cervical biopsy) will be taken and sent to the lab for analysis. Colposcopy is usually well tolerated by women, although some may feel a burning sensation during the procedure. After a cervical or vaginal biopsy, minor bleeding may occur, usually short in duration, and sexual abstinence for 3 days is recommended. Some women may experience mild, short-lasting uterine cramps. The benefit of undergoing this examination is that it is the best method for diagnosing precancerous cervical lesions. The collected materials, Pap smears, and any cervical and/or vaginal biopsies will be stored in a laboratory during the course of the study and may be used, if necessary, to confirm initial diagnoses.

By signing this form, you agree to the storage and future research use of the collected materials.
The data collected will be confidential and used solely for research purposes. This study will evaluate changes found in Pap smears that may develop into cervical cancer if left untreated. As a benefit of your participation, you will undergo tests for cervical cancer-related factors not routinely available in the public healthcare system.

If any medical issues directly related to your participation in this study arise, you are guaranteed treatment for these issues and may request compensation for any harm resulting from participation in the study. If you agree to participate, you will receive a signed copy of this informed consent form.

You will have access to the researchers responsible for the study should you have any questions. The principal investigator is Dr. Cristina Paula Castanheira, supported by Prof. Gustavo A.R. Maciel. They can be reached at:

**Av. Dr. Enéas Carvalho de Aguiar, 255 – Central Institute, Hospital das Clínicas, 10th floor – São Paulo, ZIP Code: 05403-000. Phone: +55-11-2661-7621.**

If you have any concerns or questions about the ethical aspects of the research, contact the Research Ethics Committee (CapPesq) – **Rua Ovídio Pires de Campos, 225 – 5th floor – Phone: 2661-7585 or via email: cappesq.adm@hc.fm.usp.br**

You will not be charged for the treatment, including exams and consultations, provided during the study. Your participation is entirely voluntary (i.e., not mandatory), and you may withdraw at any time. Your decision not to participate or to withdraw will not affect your current or future medical care at this Institution. However, if you decide to withdraw, you must immediately notify the physician overseeing your care. This is for your own safety. You should also understand that your participation may be discontinued at any time by the attending physician if deemed in your best interest. You will be informed of any new findings that may influence your decision to continue or not in this study.

After reading this document, I declare that I have been adequately informed about the study: **COMPARATIVE ANALYSIS OF THE PRESENCE OF HPV, MICROBIOTA COMPONENTS, AND GENE METHYLATION PROFILE IN CERVICAL CELLS OBTAINED BY URINE, SELF-COLLECTION, AND HEALTHCARE PROFESSIONALS.**

I have discussed the above information with the Responsible Researcher, Dr. Gustavo Arantes Rosa Maciel, or a person delegated by him, Dr. Cristina Paula Castanheira, regarding my decision to participate in this study. The objectives, procedures, potential discomforts and risks, and guarantees have been clearly explained to me.

I voluntarily agree to participate in this study, sign this consent form, and receive a copy initialed by the researcher.

__________________________________________Date:___/___/_____
**Signature of participant/legal representative**

**__________________________________________________**

**Name of participant**

__________________________________________Date:___/___/____ _
**Signature of the responsible researcher**

**Supplementary Data 2 Q**

**Acceptability Questionnaire for Urine Collection and Vaginal Self-Collection Procedures**

**IDENTIFICATION:**
**NAME:** _______________________________________________________________

**1. Regarding the instructions you received to use the urine collection device, you considered them:**

( ) very easy

( ) easy

( ) difficult

( ) very difficult

**2. Regarding the use of the urine collection device, you considered it:**

( ) very easy

( ) easy

( ) difficult

( ) very difficult

**3. Did you feel embarrassed while using the urine collection device?**

( ) not at all embarrassed

( ) slightly embarrassed

( ) moderately embarrassed

( ) very embarrassed

**4. Did you feel any discomfort while using the urine collection device?**

( ) no discomfort at all

( ) slight discomfort

( ) discomfort

( ) severe discomfort

**5. Regarding the instructions you received for using the brush for vaginal self-collection, you considered them:**

( ) very easy

( ) easy

( ) difficult

( ) very difficult

**6. Regarding the use of the brush for vaginal self-collection, you considered it:**

( ) very easy

( ) easy

( ) difficult

( ) very difficult

**7. Did you feel embarrassed while using the vaginal self-collection brush?**

( ) not at all embarrassed

( ) slightly embarrassed

( ) moderately embarrassed

( ) very embarrassed

**8. Did you feel any discomfort while using the vaginal self-collection brush?**

( ) no discomfort at all

( ) slight discomfort

( ) discomfort

( ) severe discomfort

**9. Regarding the instructions you received for the collection performed by the doctor, you considered them:**

( ) very easy

( ) easy

( ) difficult

( ) very difficult

**10. Did you feel embarrassed during the collection performed by the doctor?**

( ) not at all embarrassed

( ) slightly embarrassed

( ) moderated embarrassed

( ) very embarrassed

**11. Did you feel any discomfort during the collection performed by the doctor?**

( ) no discomfort

( ) slight discomfort

( ) discomfort

( ) severe discomfort

**12. If you could choose one of the collection methods you underwent today to perform the cervical cancer screening, which one would you choose?**

( ) urine collection device

( ) self-collected device (vaginal brush)

( ) collection by healthcare professional

( ) no preference or indifferent

**13. For what reasons? You may select more than one answer.**

( ) less discomfort or pain

( ) less embarrassment or shame

( ) greater convenience

( ) ease of performing the collection at home

( ) possibility of collecting the sample alone

( ) fear of collecting incorrectly

( ) greater confidence in collection performed by a healthcare professional

( ) indiferent- accepts all three methods

**14. Regarding the instructional video on how to use the urine collection device and the self-collection brush, what did you think?**

( ) the video helped a lot in understanding

( ) the video helped a little in understanding

( ) the video did not help in understanding

( ) the video made understanding more difficult

**15. Do you think that healthcare services should use instructional videos for certain medical procedures?**

( ) yes

( ) no

**16. For what reasons?**

( ) the video made me feel calmer and more confident about using the urine collector and brush
( ) the video made me feel confused, anxious, and/or unsure about how to use the urine collector and brush

( ) I prefer an explanation from a healthcare professional rather than watching a video
( ) I would like to watch the video and then clarify my questions with a healthcare professional
